# Distinct effects of etoposide on glutamine-addicted neuroblastoma

**DOI:** 10.1007/s00018-019-03232-z

**Published:** 2019-08-07

**Authors:** Kadri Valter, Polina Maximchik, Alibek Abdrakhmanov, Viacheslav Senichkin, Boris Zhivotovsky, Vladimir Gogvadze

**Affiliations:** 1 Division of Toxicology, Institute of Environmental Medicine, Karolinska Institutet, Box 210, 171 77 Stockholm, Sweden; 2 Faculty of Medicine, MV Lomonosov Moscow State University, 119991 Moscow, Russia

**Keywords:** Apoptosis, Neuroblastoma, Mitochondria, Oxidative stress, Respiratory chain

## Abstract

The majority of anticancer drugs are DNA-damaging agents, and whether or not they may directly target mitochondria remains unclear. In addition, tumors such as neuroblastoma exhibit addiction to glutamine in spite of it being a nonessential amino acid. Our aim was to evaluate the direct effect of widely used anticancer drugs on mitochondrial activity in combination with glutamine withdrawal, and possible apoptotic effects of such interaction. Our results revealed that etoposide inhibits mitochondrial respiratory chain Complex I causing the leakage of electrons and the superoxide radical formation. However, it was not sufficient to induce apoptosis, and apoptotic manifestation was detectable only alongside the withdrawal of glutamine, a precursor for antioxidant glutathione. Thus, the simultaneous depletion of glutathione and destabilization of mitochondria by ROS can compromise the barrier properties of the mitochondrial membrane, leading to cytochrome *c* release and the activation of the mitochondrial apoptotic pathway. Thus, the depletion of antioxidants or the inhibition of the pathways responsible for cellular antioxidant response can enhance mitochondrial targeting and strengthen antitumor therapy.

**Electronic supplementary material:**

The online version of this article (10.1007/s00018-019-03232-z) contains supplementary material, which is available to authorized users.

## Introduction

Tumor development can be counteracted by the stimulation of apoptosis or other forms of programmed cell death [1, 2]. In the majority of tumor cells, apoptotic pathways are dormant owing to the low expression of pro-apoptotic factors, such as pro-apoptotic members of Bcl-2 family proteins [3, 4], or glycolytic shift, described by Otto Warburg, which stimulates glycolysis in tumor cells and silences mitochondria [5, 6].

Mitochondria are paramount in the stimulation/execution of apoptosis. Targeting mitochondria and the stimulation of mitochondrial apoptotic pathway has proven to be a promising strategy in tumor cell elimination [7, 8]. Mitochondria can be targeted by compounds that affect mitochondrial activity and/or contribute to the outer mitochondrial membrane (OMM) permeabilization, which may lead to the activation of programmed cell death. Various conventionally used anticancer therapeutic drugs are DNA-damaging agents, but their ability to target mitochondria remains unclear.

Targeting the mitochondrial electron transport chain can influence proton gradient formation and instigate the dissipation of the mitochondrial membrane potential (∆*ψ*). Furthermore, obstructions in respiratory chain electron flow can induce electron leakage and the formation of reactive oxygen species (ROS) [9–12]. These events can cause OMM permeabilization, leading to cytochrome *c* release and the activation of apoptosis. Although mitochondria are regarded as a source of ROS, they also possess a powerful detoxifying system responsible for ROS elimination [13]. Moreover, in some studies, mitochondria are regarded as “a sink for ROS generated in other cellular compartments”, because they can also rapidly detoxify ROS [14]. Thus, the ability of mitochondria to diminish ROS constitutes an important factor regulating apoptosis induction.

In addition to glycolytic shift, some tumors display addiction to glutamine, even though this is a nonessential amino acid [15]. Attained glutamine is converted by glutaminase into glutamate, which is used as an important pre-substrate for the Krebs cycle, synthesis of glutathione, and amino and fatty acids to support intensive proliferation of cancer cells [16, 17]. One such glutamine-dependent tumor is neuroblastoma (NB), the most common solid cancer in childhood, which is often driven by oncogene MYCN amplification [18, 19]. MYCN is a transcription factor that belongs to the family of MYC oncoproteins. MYC and MYCN are involved in glutaminolysis. Thus, suppression of MYC in human glioma SF188 cells using shRNA caused the reduction in glutamine consumption and ammonia production [20]. Knockdown of MYCN inhibited glutaminolysis in NB cells, while overexpression of MYCN in neural crest progenitor cells enhanced glutaminolysis [21]. Therefore, targeting glutaminolysis might be beneficial for tumor cell elimination.

Our aim was to evaluate the direct effect of widely used anticancer drugs on mitochondrial activity in combination with glutamine withdrawal, and the possible apoptotic effects of such interaction.

## Materials and methods

### Cell lines

TET21N derived from SHEP cells possess an inducible expression system on the basis of the tetracycline repressor of *E. coli* to reversibly express MYCN in a human NB cell line [22]. TET21N cell line was cultured in 37 °C humidified air/CO_2_ (5%) atmosphere with RPMI 1640 (Sigma) complete medium including heat-inactivated fetal calf serum 10% (w/v), penicillin/streptomycin (100 U/ml), 100 µg/ml hygromycin B, and 200 µg/ml geneticin. Glutamine deprivation was achieved by changing the regular medium to glutamine-free RPMI 1640 (Sigma) 24 h prior to treatment. Switching off the oncogene MYCN was done by incubation of the cells with 0.1 μg/ml doxycycline 24 h before seeding the sample plates. HCT116 cell line was cultured in 37 °C humidified air/CO_2_ (5%) atmosphere with DMEM (Gibco^®^) complete medium including heat-inactivated fetal calf serum 10% (w/v) and penicillin/streptomycin (100 U/ml).

### Mitochondrial oxygen consumption

15,000 cells/well were seeded on 96-well plates (Seahorse Bioscience, Billerica, MA) and allowed to adhere overnight. The attached cells were washed with Seahorse assay medium and incubated in the same medium (0.175 ml) for 1 h at 37 °C. Respiration analysis began with the assessment of basal oxygen consumption rate (OCR), followed by the injection of various drugs into the wells. The compounds of interest were injected from port A, and subsequent additions from ports B to D were oligomycin, carbonyl cyanide 3-chlorophenylhydrazone (CCCP), and rotenone plus antimycin A. Injections from B to D were conducted to respectively assess phosphorylating respiration, spare respiratory capacity, and non-mitochondrial respiration. The data were normalized to the protein content of each well.

In addition, oxygen consumption was measured in cells with permeabilized plasma membrane using an oxygen electrode (Hansatech Instruments, Norfolk, UK). Cells were harvested and resuspended in KCl-based buffer, containing 5 mM KH_2_PO_4_, 1 mM MgCl_2_, 5 mM Tris (pH 7.5), and mild detergent digitonin (0.01%). The mitochondrial Complex I substrates pyruvate and malate, or Complex II substrate succinate together with rotenone to block Complex I activity, were supplemented. Measurements were analyzed using OxygraphPlus software (Hansatech Instruments, Norfolk, UK).

### Mitochondrial transmembrane potential (Δ*ψ*)

The mitochondrial transmembrane potential (Δ*ψ*) was measured once the cells had been loaded with mitochondria-accumulating fluorescent tetramethylrhodamine ethyl ester (TMRE, 25 nM, Molecular Probes^®^) dye. The cells were incubated with TMRE for 20 min at 37 °C, trypsinized, resuspended in HEPES buffer containing 25 nM TMRE, and analyzed by BD Accuri™ C6 flow cytometer.

### Mitochondrial ROS

Mitochondrial superoxide production was detected by staining cells with MitoSOX™ Red (Molecular Probes^®^) dye according to the manufacturer’s protocol and analyzed using the BD Accuri™ C6 flow cytometer.

### Alterations in cytosolic Ca^2+^

Cells were loaded with fluorescent Ca^2+^ indicator dye Fluo-4 (2 µM, 30 min, Molecular Probes^®^), and washed and incubated in indicator-free medium for 30 min. Samples were subsequently subjected to harvesting and flow cytometer analysis by BD Accuri™ C6.

### Western-blot analysis

The samples were collected, mixed with Laemmli’s loading buffer, and boiled for 10 min. The proteins of interest were separated by a 10–15% sodium dodecyl sulfate polyacrylamide gel electrophoresis (SDS-PAGE). Electroblotting to nitrocellulose was conducted for 1.5 h at 120 V, followed by 5% non-fat milk phosphate-buffered saline (PBS) blocking for 1 h at room temperature. Having been washed with PBS (3 ×, 10 min), primary antibodies were added for overnight incubation at 4 °C. The membranes were rinsed and incubated with secondary antibodies (1:7000) for 1.5 h and the blots were visualized by Licor^®^ Odyssey^®^ CLx Imaging System or X-ray film.

Cytochrome *c* release was detected once the samples were permeabilized in digitonin-containing fractionation buffer (150 mM KCl, 1 mM MgCl_2_, 0.2 mM EGTA, 0.05% digitonin, 5 mM Tris, and pH 7.5) for 15 min at RT. Following centrifugation at 13,400 rpm for 5 min, the samples were separated into supernatant and pellet fractions, mixed with Laemmli’s buffer and analyzed using the previously described immunodetection protocol.

The samples’ protein concentration was assessed by Pierce bicinchoninic acid (BCA) colorimetric assay in accordance with the manufacturer’s instructions using a VersaMax™ microplate reader. The following primary antibodies were used: cytochrome *c* (556433, BD Biosciences), PARP (11835238001, ROCHE), C-PARP (5625, Cell Signaling), GLS1 (ab156876, Abcam), GLS2 (ab113509, Abcam), p53 (sc-126, Santa Cruz), BID (611528, BD Biosciences), and GAPDH (Trevigen).

### Measurement of caspase-3-like activity

The cells were harvested and resuspended in PBS at RT. 15 µl of the suspension were added in triplicate to 96-well plate and frozen at − 20 °C. The remaining suspension was used for protein concentration analysis by BCA protein assay. For caspase-3-like activity assay, 50 µl of peptide substrate buffer (100 mM HEPES, 10% sucrose, 0.1% Chaps, 5 mM DTT, 0.001% NP-40, and 40 µM DEVD-AMC) was added into each well of the thawed samples. Caspase-3-like activity was measured by detecting the caspase-3 mediated cleavage of DEVD-AMC and the release of fluorogenic 7-amino-4-methylcoumarin (AMC) in a fluorometric assay. Data were obtained using a Fluoroscan II plate reader (Labsystems), with excitation at 355-nm and emission at 460-nm wavelengths. The fluorescence results were normalized by protein content.

### Measurement of glutathione content

The content of glutathione in aliquots of cells was assessed as soluble SH groups [23] after precipitation of proteins by equal amount of 5% trichloroacetic acid, followed by centrifugation at 6500×*g* for 5 min. The supernatant was transferred into a 96-well plate, 100 μl of 0.5 M Tris buffer (pH 8.9) and 20 μl of DTNB were added and incubated for 10 min. The absorbance was measured at 412 nm using a VersaMax™ microplate reader.

### Statistics

All of the data presented were based on at least three repetitions and represent the average results. The error bars represent SD. Statistical significance was assessed by Student’s *t* test (two-tailed distribution, two-sample unequal variance) or two-way ANOVA (*p < 0.05 and **p < 0.01).

## Results

### The effect of anticancer drugs on oxygen consumption rate (OCR)

**Fig. 1:**
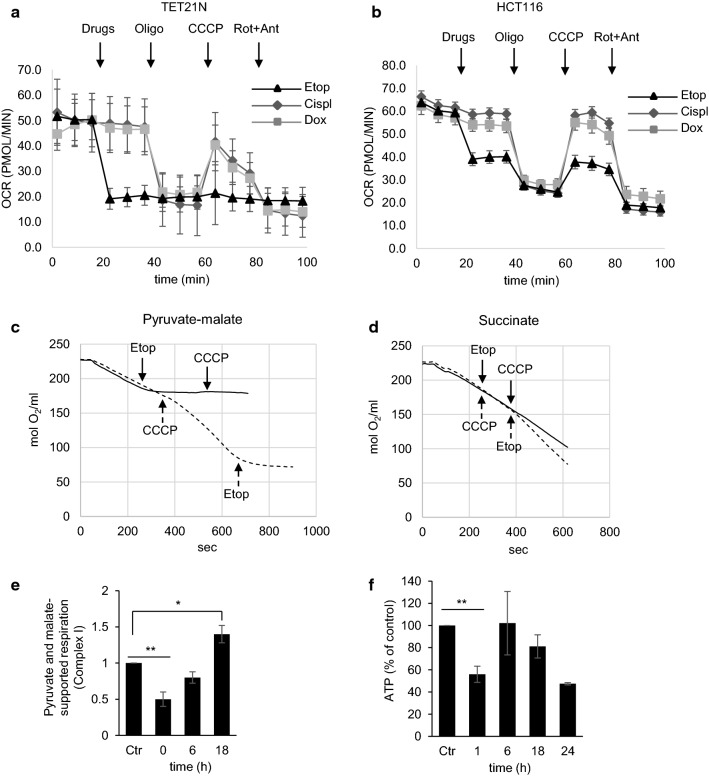
The effect of various anticancer drugs on oxygen consumption rate (OCR). **a** Assessment of the immediate effect of anticancer drugs on oxygen consumption in TET21N and HCT116 (**b**) cells. Oligomycin (Oligo) was used to analyze phosphorylating respiration, the uncoupler CCCP to assess spare respiratory capacity, and rotenone (Rot) plus antimycin (Ant) to estimate non-mitochondrial oxygen consumption. *Etop* etoposide, *Cisp* cisplatin, *Dox* doxorubicin; **c** immediate effect of etoposide (34 µM) on respiration, supported by pyruvate and malate (Complex I), or succinate (Complex II) (**d**). Solid line corresponds to solid arrows and dotted line to dotted arrows indicating the order of drug administration; **e** oxygen consumption supported by Complex I substrates after 0 h, 6 h, and 18 h of treatment with etoposide (34 µM). The experiments **c**–**e** were performed using cell with digitonin-permeabilized plasma membrane; **f** assessment of ATP content at different time points in TET21N cells treated with etoposide (34 µM). *p < 0.05, **p < 0.01

To evaluate the immediate effect of anticancer drugs on mitochondrial functions, assessment of the oxygen consumption rate (OCR) was performed in TET21N cells. After evaluation of basal respiration, cisplatin, doxorubicin, or etoposide was injected into the wells. Neither cisplatin nor doxorubicin had an immediate effect on the respiration rate, whereas etoposide caused a rapid decrease in OCR (Fig. 1a). Figure 1a represents a typical mitochondrial OCR test. After recording the basal oxygen consumption, oligomycin was used to analyze phosphorylating respiration, the uncoupler carbonyl cyanide m-chlorophenylhydrazone (CCCP) to assess the spare respiratory capacity, and rotenone *plus* antimycin to measure non-mitochondrial oxygen consumption. The suppression of respiration by etoposide was observed not only in TET21N cells, but also in HCT116 colon carcinoma cell line (Fig. 1b). Thus, the inhibition of respiration by etoposide is a common consequence for various cell lines. Assessment of OCR in TET21N cells after 6 and 18 h of etoposide treatment revealed its decrease as the apoptosis progressed (Supplementary Fig. 1a, b).

### The effect of etoposide on the respiratory complexes

To elucidate which complexes of the mitochondrial respiratory chain are affected by etoposide, oxygen consumption was evaluated in cells with digitonin-permeabilized plasma membrane, enabling mitochondria to be provided with substrates for Complex I (pyruvate and malate) or Complex II (succinate). The results obtained revealed an immediate and severe suppression of Complex I upon the addition of etoposide both before (solid line) and after (dashed line) mitochondrial uncoupling with the protonophore CCCP (Fig. 1c). At the same time, no significant instantaneous effect on the Complex II activity after adding etoposide was detected (Fig. 1d). The assessment of the continuous etoposide treatment (6 h and 18 h) revealed a restoration of Complex I activity to initial level after 6 h of incubation with etoposide (Fig. 1e). The suppression of oxygen consumption correlated with the level of ATP (Fig. 1f). The initial decrease in ATP level, which observed 1 h after etoposide administration, was followed by its restoration after 6 h. After 18 h, the content of ATP was decreased again, apparently because of progressing cell death.

### The effect of etoposide on cellular Ca^2+^ content

**Fig. 2:**
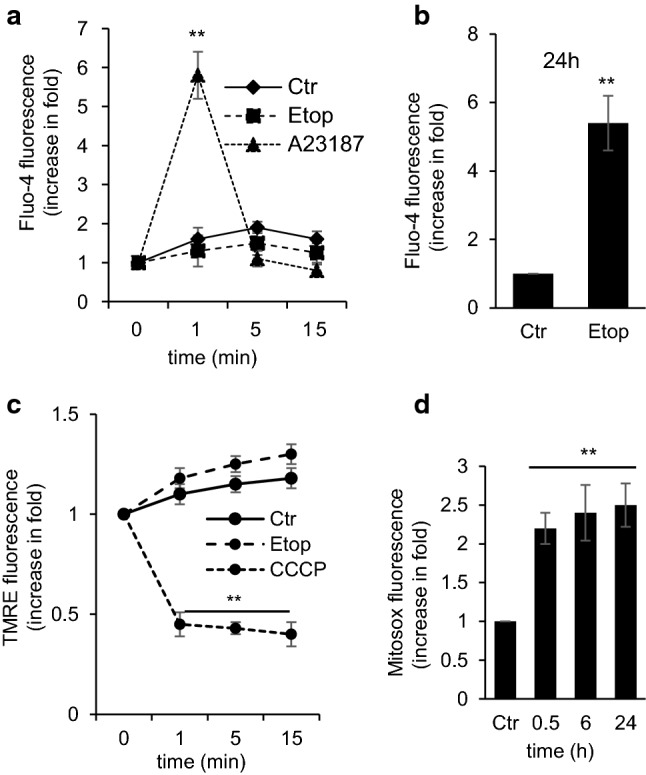
The effect of etoposide on cellular Ca^2+^ content, mitochondrial ∆*ψ*, and superoxide production in TET21N cells analyzed by flow cytometry. **a** Ca^2+^ content after etoposide (34 µM) addition detected with Fluo-4 dye, A23187 (0.1 µM) used as a positive control; **b** Ca^2+^ content after treatment with etoposide (34 µM) for 24 h, detected with Fluo-4; **c** mitochondrial ∆*ψ* after etoposide (34 µM) addition detected with TMRE staining, CCCP used as a negative control; **d** superoxide production after 0.5 h, 6 h, and 24 h of treatment with etoposide (34 µM) detected with superoxide-sensitive dye MitoSox™ Red. *p < 0.05, **p < 0.01

The direct effect of etoposide on respiration and the deterioration of mitochondria may compromise cellular Ca^2+^ homeostasis and trigger pathological processes [24, 25]. To answer the question whether etoposide can affect cellular Ca^2+^ homeostasis, the level of cytosolic Ca^2+^ was assessed using the fluorescent Ca^2+^ indicator dye Fluo-4. The results did not detect any immediate effect of etoposide on the cytosolic Ca^2+^ level (Fig. 2a). However, elevation of the cytosolic Ca^2+^ was observed after 24 h treatment with etoposide (Fig. 2b), apparently was due to the processes related to cell death execution. As positive control, 0.1 µM of ionophore A23187 was used. The ionophore caused a transient increase of intracellular Ca^2+^ (Fig. 2a, dotted line).

### The effect of etoposide on mitochondrial ∆*ψ* and superoxide production

Inhibition of the respiratory chain can compromise proton gradient formation, which will lead to attenuation of the mitochondrial ∆*ψ*. Assessment of ∆*ψ* in TET21N cells was performed by staining cells with cationic fluorescent dye TMRE (tetramethylrhodamine ethyl ester), which accumulates in energized mitochondria. The measurements demonstrated that etoposide’s suppression of the respiratory chain has a minor impact on ∆*ψ* (Fig. 2c). Nevertheless, the obstruction of Complex I in combination with changes in ∆*ψ* can cause a leakage of electrons and the formation of superoxide radical [26]. To test this possibility, the assessment of mitochondrial superoxide production following etoposide administration was performed. The addition of etoposide to TET21N cells triggered ROS formation as early as 0.5 h after administration, which remained exalted after 6 h and 24 h (Fig. 2d).

### The effect of glutamine removal on etoposide-induced apoptosis in TET21N cells

**Fig. 3:**
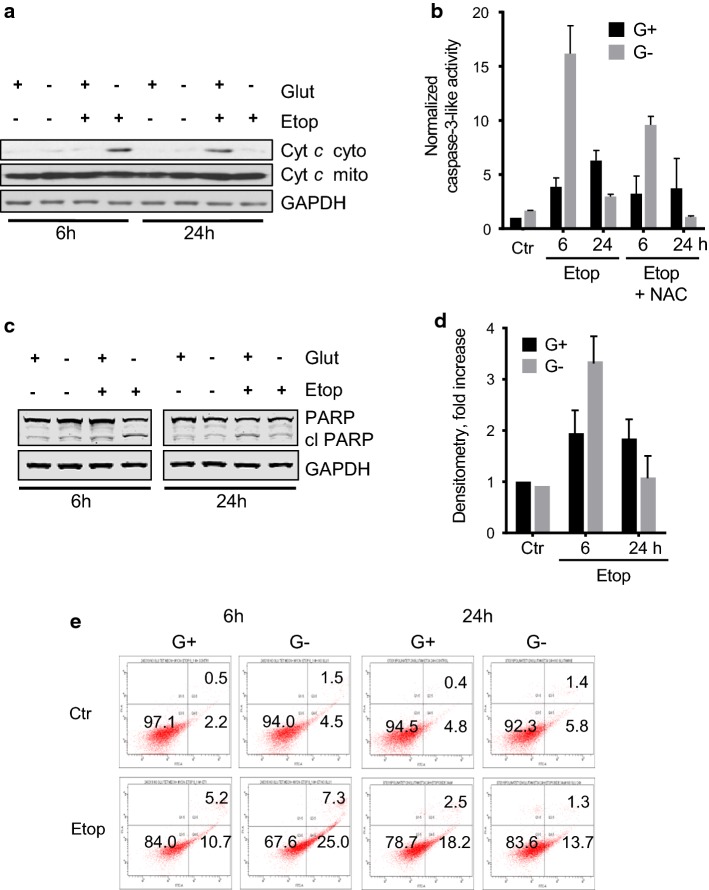
The effect of glutamine removal on etoposide-induced apoptosis in TET21N cells. **a** cytochrome *c* release after 6 and 24 h of etoposide treatment in the presence and absence of glutamine; **b** caspase-3-like activity in glutamine-supported and -deprived cells after 6 h and 24 h treatment with etoposide and the protective effect of the antioxidant *N*-acetylcysteine (NAC); **c** cleavage of PARP in glutamine-supported and glutamine-deprived cells after 6 h and 24 h of treatment with etoposide and densitometry of the corresponding blot (**d**); **e** phosphatidylserine externalization in cells treated with etoposide (34 μM) in the presence or absence of glutamine after 6 and 24 h of incubation

The suppression of mitochondrial respiration and stimulation of ROS production can potentially lead to mitochondrial deterioration and triggering the mitochondrial apoptotic pathway. However, we could detect only relatively modest manifestations of apoptosis after 6 h of etoposide treatment (Fig. 3a–e). Although mitochondria are regarded as a source of ROS, they also possess a powerful detoxifying system responsible for the elimination of oxygen radicals. One of the most important cellular endogenous antioxidants is the tripeptide glutathione. Therefore, reducing glutathione levels may enhance the deleterious effects of ROS and compromise the barrier properties of mitochondrial membranes, leading to cytochrome *c* release and the activation of the mitochondrial apoptotic pathway. Given that the cells in our experiment were grown in the presence of glutamine, a precursor of glutathione, and mitochondrial substrate of α-ketoglutarate [17], glutamine withdrawal may attenuate the level of the antioxidant and stimulate etoposide-induced apoptosis when the respiratory chain is blocked and ROS production is triggered. To test this possibility, TET21N cells were cultured for 24 h in the presence or absence of glutamine and treated subsequently with etoposide for 6 h and 24 h (the experiment scheme is shown in Supplementary Fig. 2), followed by the analysis of several manifestations of apoptosis.

The results obtained with glutamine-deprived cells after 6 h of exposure to etoposide indeed revealed the stimulation of cell death assessed by cytochrome *c* release from mitochondria (Fig. 3a), caspase-3-like activity (Fig. 3b), cleavage of PARP (Fig. 3c, d), and the appearance of phosphatidylserine on the outer leaflet of plasma membrane tested with staining with Annexin V and propidium iodide (PI) (Fig. 3e). Withdrawal of glutamine markedly stimulated apoptosis assessed by all these parameters. The cell death stimulation detected in glutamine-deprived cells was dependent on ROS, because the antioxidant *N*-acetylcysteine significantly suppressed caspase-3-like activity in glutamine-deprived samples (Fig. 3b). A similar result was obtained with another antioxidant trolox, a water-soluble analog of vitamin E, after both 6 and 24 h of treatment with etoposide (Supplementary Fig. 3). After 24 h of etoposide treatment, as we have shown earlier [27], withdrawal of glutamine suppressed etoposide-induced apoptosis (Fig. 3a–e).

**Fig. 4:**
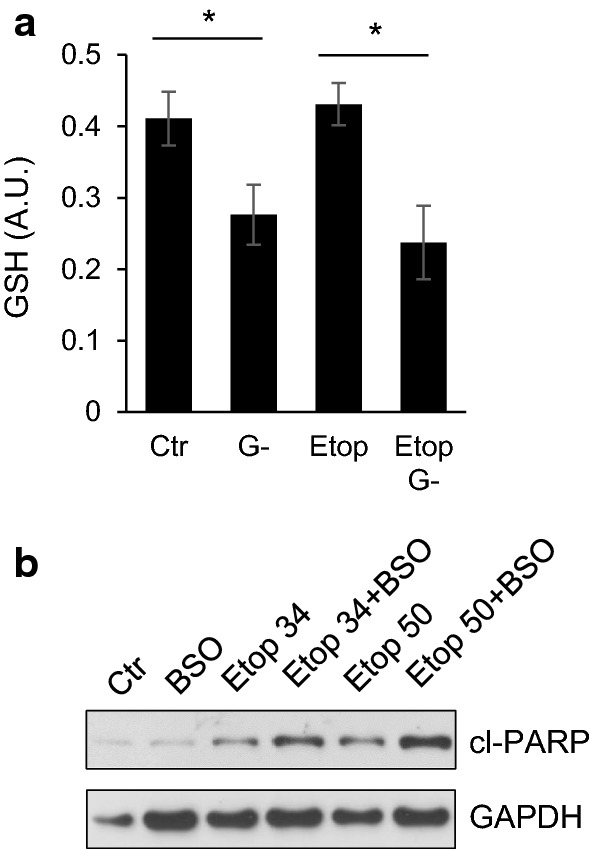
Involvement of glutathione in apoptotic response of TET21N cells. **a** The level of glutathione in TET21N cells treated with etoposide (34 µM) for 6 h in the presence and absence of glutamine, *p < 0.05. **b** glutathione depletion by 200 nM buthionine sulfoximine (BSO) stimulates etoposide-induced apoptosis

To test whether cell death stimulation is a consequence of impaired antioxidant defense, the level of glutathione was assessed after removal of glutamine. Indeed, glutamine deprivation for 6 h attenuated the glutathione content (Fig. 4a) both in control- and etoposide-treated cells. To prove the importance of antioxidant defense in apoptosis progression, depletion of glutathione was performed using buthionine sulfoximine (BSO). BSO stimulated cell death triggered by 34 and 50 μM etoposide assessed by PARP cleavage (Fig. 4b).

### The effect of glutamine removal on etoposide-induced apoptosis in MYCN-deficient cells

**Fig. 5:**
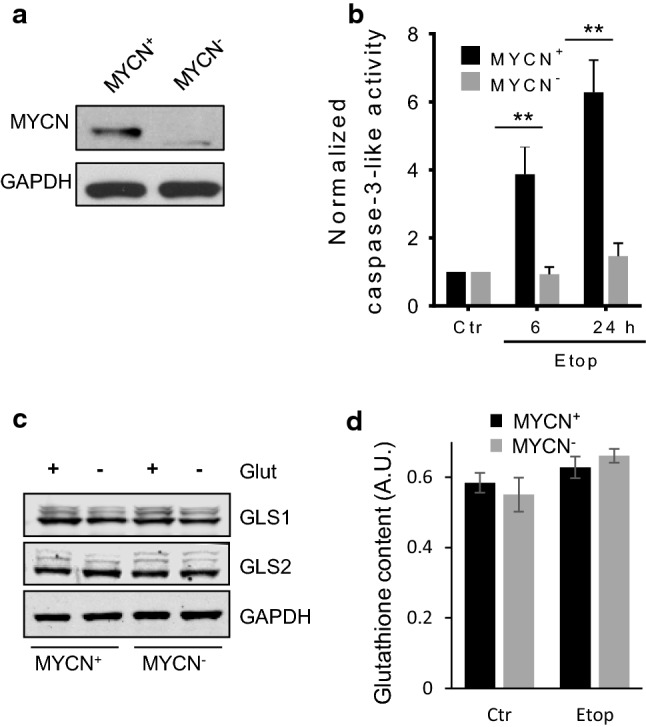
The effect of etoposide on MYCN^+^ and MYCN^−^ TET21N cells. **a** The level of MYCN in TET21N cells without and with doxycycline treatment; **b** caspase-3-like activity in MYCN^+^ and MYCN^−^ TET21N cells after 6 h and 24 h of etoposide treatment; **c** GLS1 and GLS2 expression in glutamine-supported and -deprived MYCN^+^ and MYCN^−^ TET21N cells; **d** glutathione content in MYCN^+^ and MYCN^−^ TET21N cells treated with etoposide for 6 h. Concentration of etoposide—34 µM; *p < 0.05, **p < 0.01

Glutamine is converted into glutamate, a precursor of glutathione, by the enzyme glutaminase. This enzyme is a target of oncogene MYCN, and downregulation of MYCN expression should attenuate the level of glutathione and subsequently enhance the sensitivity of cells to ROS. Thus, the consequence of “switching off” MYCN before etoposide treatment was analyzed. Incubation of TET21N cells with doxycycline for 24 h downregulated MYCN expression completely (Fig. 5a). Surprisingly, in comparison to the MYCN-amplified cells (MYCN^+^), the absence of MYCN (MYCN^−^ cells) markedly suppressed etoposide-induced apoptosis both after 6 h and 24 h of etoposide treatment, verified by caspase-3-like activity (Fig. 5b) and phosphatidylserine externalization after staining with Annexin V and PI (Supplementary Fig. 4). Removal of glutamine stimulated apoptosis in MCYN^−^ cells as it has been shown for MYCN^+^ cells (Supplementary Fig. 4). Therefore, further analysis was performed to reveal the mechanisms behind this finding.

Unexpectedly, the comparison of glutaminase 1 and 2 (GLS1/2) expression in MYCN^+^ and MYCN^−^ cells did not reveal any difference (Fig. 5c). In addition, there was no decrease in the content of glutathione. In MYCN^−^ TET21N cells, a modest non-significant increase in glutathione level was observed after 6 h incubation with etoposide (Fig. 5d).

**Fig. 6:**
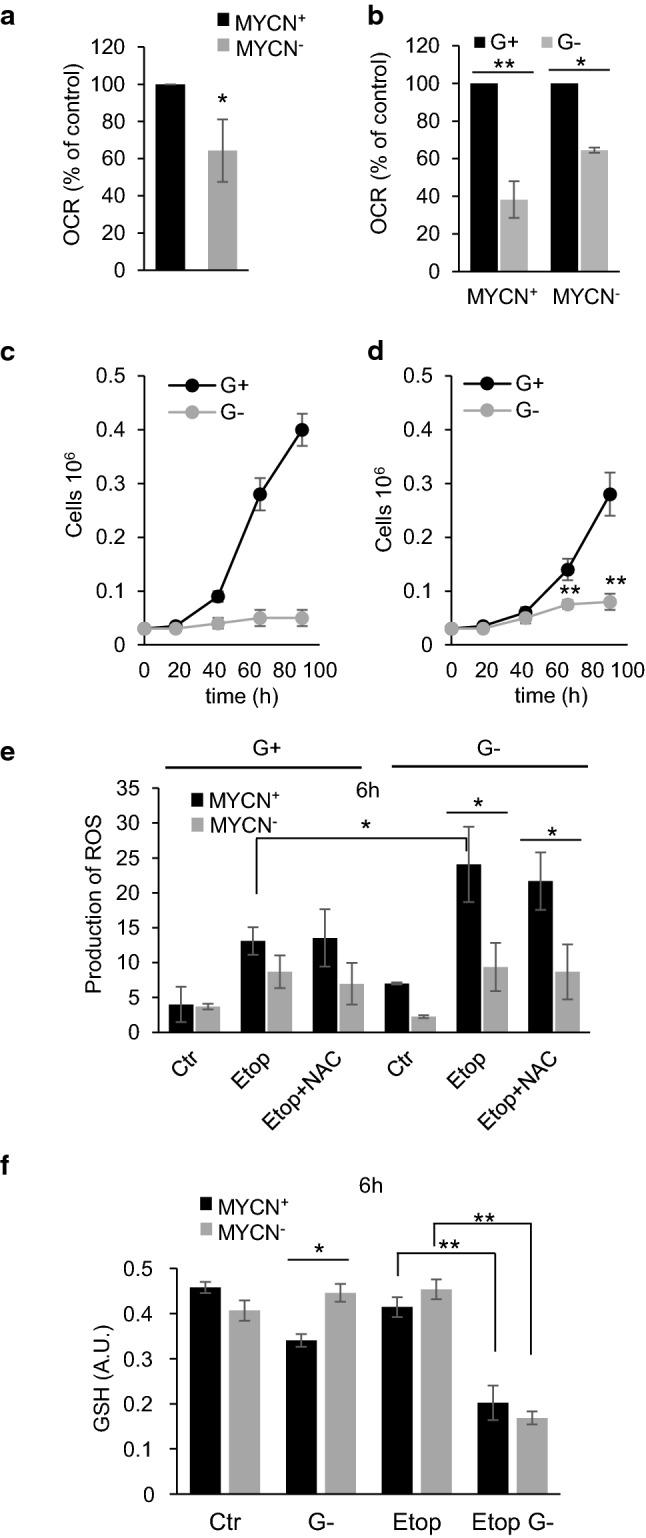
The comparison of the effect of glutamine removal on MYCN^+^ and MYCN^−^ TET21N cells: **a** switching off MYCN decreases OCR in TET21N cells; **b** attenuation of OCR after glutamine removal in MYCN^+^ and MYCN^−^ TET21N cells in percentage to control. OCR was calculated as the difference between mitochondrial oxygen consumption uncoupled by CCCP (maximal) and non-mitochondrial respiration, after inhibiting respiratory chain with rotenone *plus* antimycin; **c** MYCN^+^ TET21N cell proliferation in the presence and absence of glutamine; **d** MYCN^−^ TET21N cell proliferation in the presence and absence of glutamine; e, stimulation of superoxide radical production in etoposide-treated MYCN^+^ and MYCN^−^ TET21N cells assessed by flow cytometry using superoxide-sensitive dye MitoSox™ Red in the presence and absence of glutamine, and the effect of antioxidant NAC on this process; f, glutathione content in MYCN^+^ and MYCN^−^ TET21N cells in the presence and absence of glutamine treated with etoposide for 6 h. Concentration of etoposide—34 µM; *p < 0.05, **p < 0.01

Analysis of oxygen consumption in MYCN^+^ and MYCN^−^ TET21N cells revealed that switching MYCN off significantly decreased OCR in TET21N cells (Fig. 6a) and markedly altered respiratory response to glutamine deprivation (Fig. 6b). Thus, in MYCN^+^ cells, withdrawal of glutamine suppressed OCR by 60–70%, whereas in MYCN^−^ cells, which are supposedly less addicted to glutamine, OCR inhibition was not so prominent (30–35%) (Fig. 6c). Treatment of MYCN^−^ TET21N cells with etoposide, revealed a similar ATP decrease–increase–decrease pattern (Supplementary Fig. 5a) as already shown for MYCN^+^ cells in Fig. 1f. Glutamine deprivation decreased the level of ATP in both MYCN^+^ and MYCN^−^ TET21N cells. Etoposide treatment for 24 h further decreased the ATP content, although the difference between MYCN^+^ and MYCN^−^ TET21N cells was insignificant (Supplementary Fig. 5b). Furthermore, in MYCN^+^ TET21N cells, glutamine withdrawal prominently hampered cell proliferation (Fig. 6c). In contrast, MYCN^−^ TET21N cells, which demonstrated only partial OCR suppression after glutamine withdrawal, were able to proliferate further, although the intensity of cell division was restricted (Fig. 6d).

Apparently, due to the lower rate of oxygen consumption, superoxide production in MYCN^−^ cells upon treatment with etoposide was diminished as compared to MYCN^+^ TET21N cells (Fig. 6e). Glutamine deprivation stimulated superoxide production in MYCN^+^ TET21N cells and to a lesser extent in MYCN^−^ cells. Antioxidant NAC did not affect superoxide production neither in MYCN^+^ nor in MYCN^−^ TET21N cells irrespectively of the presence of glutamine. Removal of glutamine decreased the level of glutathione in MYCN^+^  TET21N cells, but had no effect in MYCN^−^ cells (Fig. 6e). Treatment of glutamine-deprived cells with etoposide attenuated the level of glutathione in MYCN^+^ and MYCN^−^ TET21N cells to the same extent.

In addition, we analyzed apoptotic response upon glutamine withdrawal in other cell lines with different levels of MYCN expression, in particular, MYCN expressing SK-N-BE(2) cells, and SH-SY5Y cells, in which expression of MYCN is low (Supplementary Fig. 6a). In SK-SN-BE(2) cells, glutamine withdrawal stimulated apoptosis after 6 h of treatment, but attenuated after 24 h, similarly to TET21 MYCN^+^  cells (Supplementary Fig. 6b). At the same time, death of SH-SY5Y cells did not show any dependence on glutamine. It should be mentioned, however, that these cells were far more sensitive to etoposide; ten times lower concentration of the drug was sufficient to induce death of SH-SY5Y cells.

## Discussion

The majority of anticancer drugs are aimed at nuclear DNA for tumor cell elimination. Cell death is activated when DNA damage stabilizes the transcriptional factor p53, which regulates the expression of various pro-apoptotic proteins involved in OMM permeabilization and the release of mitochondrial pro-apoptotic factors. Pro-apoptotic, BH3-only Bcl-2 family proteins, such as Bax, Puma, Noxa, and possibly Bid, are direct transcriptional targets of p53. In addition, p53 may also act in a transcription-independent manner through binding to pro-apoptotic Bcl-2-family proteins, leading to permeabilization of mitochondria. p53, itself, may also act like a BH3-only protein and antagonize Bcl-2 function (reviewed in [28]). However, the question whether anticancer drugs can directly affect mitochondria has yet to be thoroughly investigated. We found that among conventionally used anticancer drugs, only etoposide was able to target mitochondria directly. Etoposide suppressed Complex I of the mitochondrial respiratory chain (Fig. 1c), causing the leakage of the electrons, and formation of superoxide radicals (Fig. 2d).

**Fig. 7:**
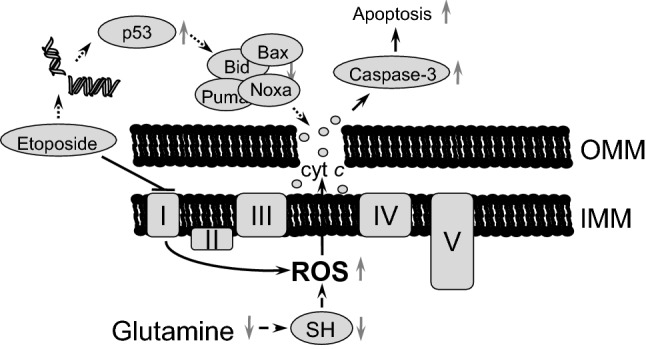
Schematic representation of possible pathways involving etoposide treatment and glutamine deprivation in TET21N cells. Etoposide has an immediate effect (black arrows) on mitochondrial respiratory chain Complex I that triggers ROS production, cytochrome *c* release and modest apoptosis induction. This effect can be increased by glutamine deprivation (dashed arrows). Long-term (24 h) etoposide treatment (dotted arrows) leads to DNA damage-dependent p53 stabilization and OMM permeabilization. *OMM* outer mitochondrial membrane; *IMM* inner mitochondrial membrane

Stimulation of oxidative stress is an important tool for tumor cell death initiation. Elevated levels of ROS have been noticed in the majority of cancers, where they facilitate numerous aspects of tumor development, progression, and metastases [13]. At the same time, tumors demonstrate higher levels of ROS-detoxifying enzymes relative to normal cells. This is necessary to prevent ROS-mediated activation of death-inducing pathways [29]. Therefore, depletion of water- and lipid-soluble antioxidants in cancer cells would allow for the accumulation of excessive amounts of ROS to the levels that initiate toxicity and reduce tumorigenesis [30]. Although elevated ROS production can trigger mitochondrial deterioration and apoptosis in tumor cells [31], alterations in the mitochondrial respiratory chain after 6 h of etoposide treatment induced only modest cell death (Fig. 3a–e). The manifestations of various characteristic features of apoptosis after 6 h of treatment only became evident upon the withdrawal of glutamine (Fig. 3a–e) that was mediated by the decrease in the content of the antioxidant glutathione (Fig. 4a). Depletion of glutathione by BSO enhanced etoposide-induced apoptosis (Fig. 4b). The suppression of apoptosis upon glutamine withdrawal 24 h after etoposide treatment (Fig. 3a–e), as we have shown earlier, correlates with downregulation of p53 in these cells [27]. A scheme representing these different pathways and their relationships is shown in Fig. 7. Apoptosis induced by etoposide is a complex process involving several factors. In a traditional model, etoposide induces DNA damage with the subsequent stabilization of p53, and triggers p53-dependent apoptosis. Previously observed suppression of apoptosis upon 24 h of treatment with etoposide and glutamine withdrawal was linked to the downregulation of p53 level, a target of MYCN [27]. At the same time, etoposide also suppresses Complex I of the mitochondrial respiratory chain and induces the production of ROS. However, the powerful mitochondrial antioxidant defense system protects cells from excessive ROS and prevents cell death. In contrast, glutamine deprivation and subsequent glutathione depletion may lead to the activation of apoptotic pathways and potentiate tumor cell elimination.

Glutamine metabolism is known to be regulated by the oncogene MYC and MYCN, which, among a number of target genes, regulates glutaminase, the enzyme that converts glutamine into glutamate, a precursor of glutathione [20, 21]. Downregulation of MYCN in NB cells was expected to reduce the level of glutaminase, and subsequently glutathione, and sensitize cells to treatment. However, contrary to expectations, these cells were more resistant to apoptosis stimulation (Fig. 5b and Supplementary Fig. 4). We have shown previously that the resistance of TET21N cells lacking MYCN can be explained by lower level of transcription factor p53, a target of MYCN, which is responsible for the expression of various pro-apoptotic members of Bcl-2 family proteins [32]. Indeed, MYCN was shown to transcriptionally regulate p53 expression [33], leading to the hypothesis that p53 upregulation might be involved in MYCN-dependent sensitization to apoptosis [34]. Apparently, the absence of MYCN can make TET21N cells less sensitive to etoposide-mediated inhibition of Complex I. It has been shown that MYCN is involved in mitochondrial biogenesis [35]; moreover, inhibition of MYCN can cause respiratory chain impairment [36] resulting in lower oxygen consumption. Indeed, in our experiments, switching MYCN off suppressed OCR (Fig. 6a). Consequently, this should lessen the leakage of electrons upon inhibition of Complex I by etoposide, and formation of superoxide radical will be attenuated as compared to MYCN^+^ TET21N cells (Fig. 6e).

Stimulation of ROS production via inhibition of various complexes of the mitochondrial respiratory chain can trigger apoptosis in tumor cells of various origins. Thus, rotenone-stimulated and antioxidant preventable HL-60 cell death, resulted from mitochondrial ROS production, was documented by various parameters, such as DNA fragmentation, cytochrome c release, and caspase 3 activity [37]. Suppression of Complex II by non-toxic doses of thenoyltrifluoroacetone, an inhibitor of Complex II, markedly augmented cell death induced by low doses of cisplatin [38]. Similarly, inhibition of Complex III by antimycin triggered apoptosis in PC12 cells [39]. The importance of the inhibition of respiratory chain complexes for apoptosis induction was further recognized with the finding that tumor necrosis factor (TNF)-α could inhibit Complex I of the mitochondrial respiratory chain [40].

Thus, the depletion of antioxidants or inhibition of pathways responsible for cellular antioxidant response can increase the efficiency of mitochondria targeting, enhance and strengthen antitumor therapy.

## Electronic supplementary material

Below is the link to the electronic supplementary material.

Supplementary material 1 (PDF 911 kb)

Supplementary material 2 (DOCX 11 kb)
