# Supplementary material for: Distinct effects of etoposide on glutamine-addicted neuroblastoma
Source: Cell Mol Life Sci. 2019 Aug 7;77(6):1197–207. doi: 10.1007/s00018-019-03232-z (PMC7109159; doi:10.1007/s00018-019-03232-z)
Supplement: Supplementary file 1 — Supplementary material 1 (PDF 911 kb) [file 18_2019_3232_MOESM1_ESM.pdf]

a

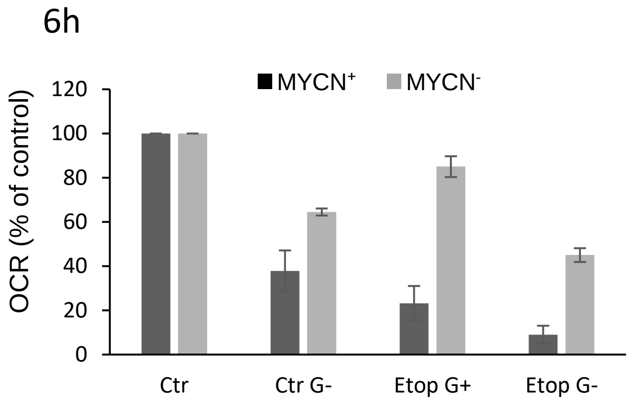

b

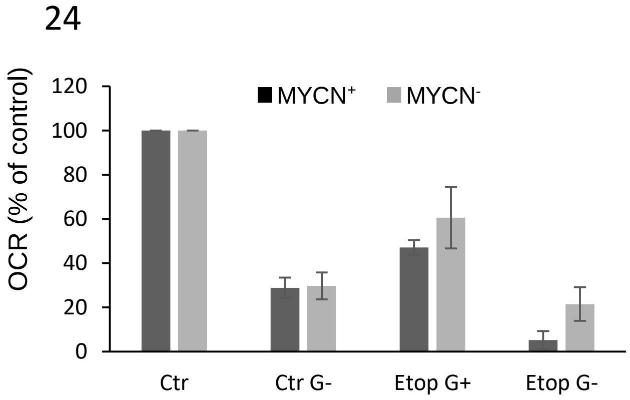

Supplementary Fig. 1

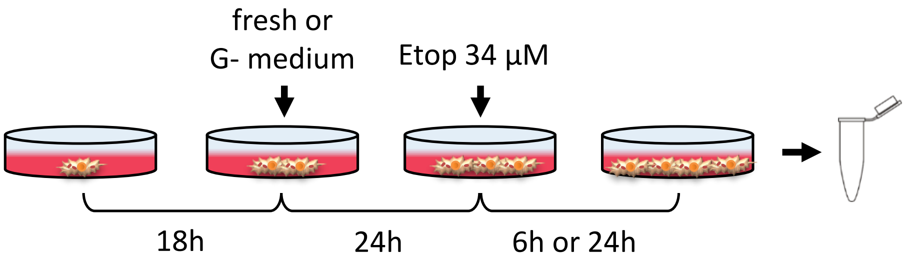

Supplementary Fig. 2

a

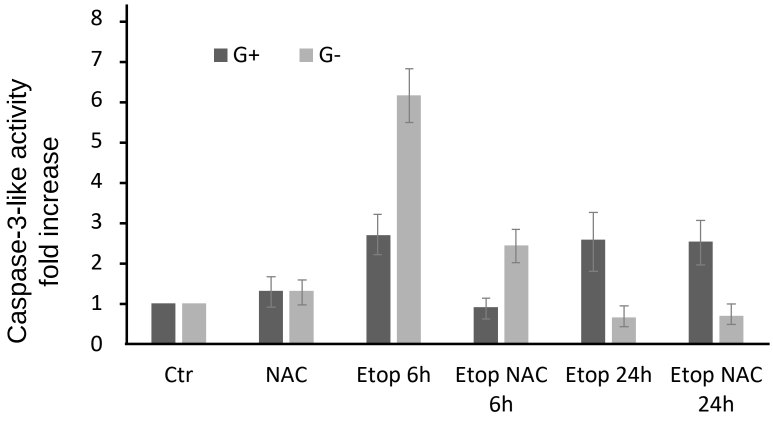

b

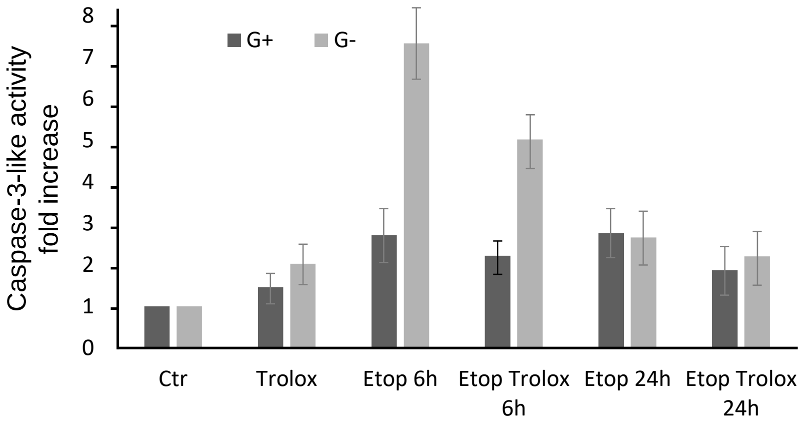

Supplementary Fig. 3

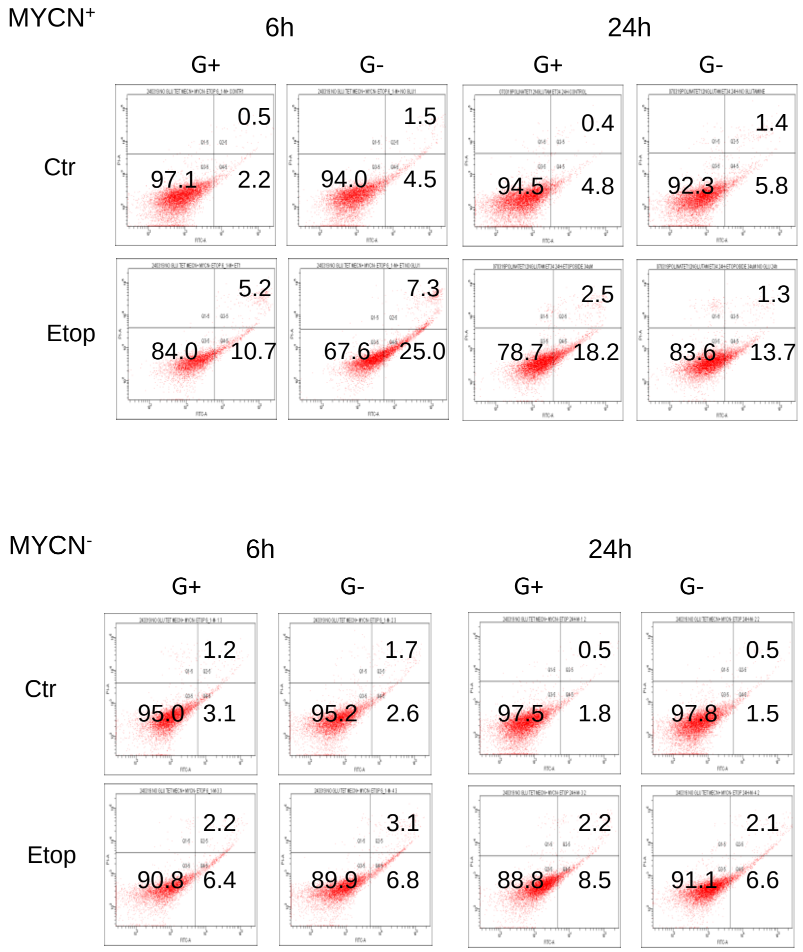

Supplementary Fig. 4

a

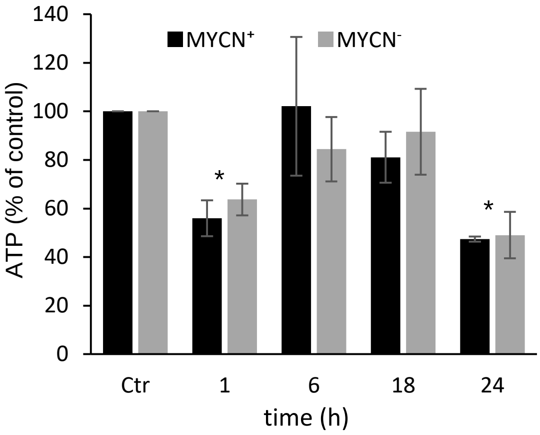

b

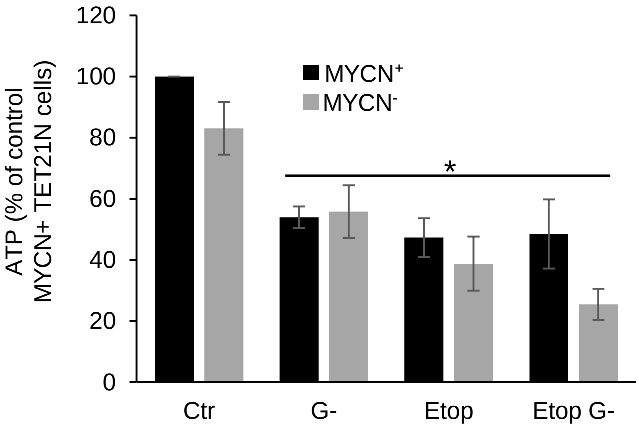

Supplementary Fig. 5

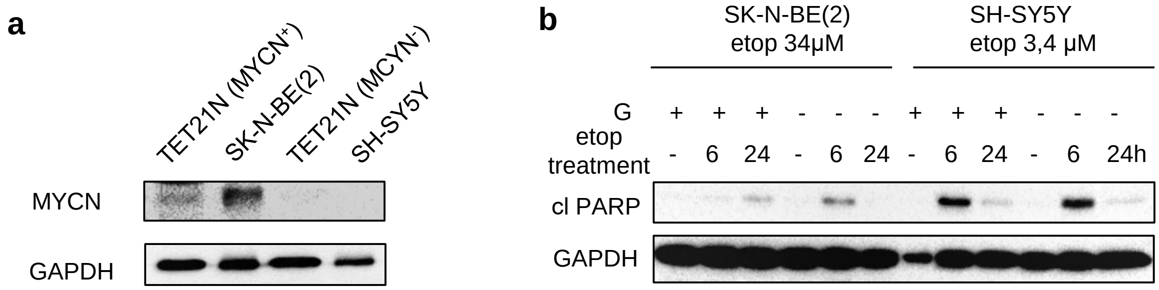

Supplementary Fig. 6
