# Supplementary material for: Distinct effects of etoposide on glutamine-addicted neuroblastoma
Source: Cell Mol Life Sci. 2019 Aug 7;77(6):1197–207. doi: 10.1007/s00018-019-03232-z (PMC7109159; doi:10.1007/s00018-019-03232-z)
Supplement: Supplementary file 2 — Supplementary material 2 (DOCX 11 kb) [file 18_2019_3232_MOESM2_ESM.docx]

**Supplementary Fig. 1.** Assessment of OCR at 6 (**a**) and 18h (**b**) after exposure to etoposide. OCR was calculated as the difference between mitochondrial respiration uncoupled by CCCP (maximal) and non-mitochondrial oxygen consumption after inhibiting mitochondrial respiration by rotenone plus antimycin (minimal) respiration. Data normalized to control.

**Supplementary Fig. 2.** Glutamine deprivation was achieved by changing the regular medium to glutamine-free RPMI 1640 (Sigma) 24h prior to treatment. Cells were cultured for 24h in the presence or absence of glutamine and subsequently treated with etoposide for 6h and 24h.

**Supplementary Fig. 3**. Antioxidants reverse etoposide-induced apoptosis stimulation caused by glutamine deprivation, **a** – NAC, **b** - trolox. Concentration of etoposide - 34µM.

**Supplementary Fig.4.** Phosphatidylserine exposure assessed by flow cytometry after staining with Annexin V and PI, in MYCN^+^ and MYCN^-^ TET21N cells treated with etoposide (34 μM) for 6 and 24h in the presence and absence of glutamine.

**Supplementary Fig. 5.** Assessment of ATP content in MYCN^+^ and MYCN^-^ TET21N cells. a, time-dependency of ATP content in MYCN^+^ and MYCN^-^ TET21N cells treated with 34µM etoposide. ATP content in MYCN^+^ and MYCN^-^ TET21N cells treated with etoposide in the presence and absence of glutamine. p<0.05.

**Supplementary Fig. 6.** MYCN content and sensitivity of various neuroblastoma cells to glutamine withdrawal. **a,** MYCN content in various neuroblastoma cell lines; **b,** apoptotic response in the presence and absence of glutamine assessed after 6 and 24 h of incubation. Concentration of etoposide - 34µM for SK-N-BE(2) cells and 3,4µM for SH-SY5Y cells.
